# Time-Dependent Effects of Training on Cardiovascular Control in Spontaneously Hypertensive Rats: Role for Brain Oxidative Stress and Inflammation and Baroreflex Sensitivity

**DOI:** 10.1371/journal.pone.0094927

**Published:** 2014-05-01

**Authors:** Gustavo S. Masson, Tassia S. R. Costa, Lidia Yshii, Denise C. Fernandes, Pedro Paulo Silva Soares, Francisco R. Laurindo, Cristoforo Scavone, Lisete C. Michelini

**Affiliations:** 1 Department of Physiology and Biophysics, Institute of Biomedical Sciences, University of Sao Paulo, Sao Paulo, Brazil; 2 Department of Pharmacology, Institute of Biomedical Sciences, University of Sao Paulo, Sao Paulo, Brazil; 3 Vascular Biology Laboratory, Heart Institute, University of Sao Paulo, Sao Paulo, Brazil; 4 Department of Physiology & Pharmacology, Fluminense Federal University, Niterói, Brazil; University of São Paulo, Brazil

## Abstract

Baroreflex dysfunction, oxidative stress and inflammation, important hallmarks of hypertension, are attenuated by exercise training. In this study, we investigated the relationships and time-course changes of cardiovascular parameters, pro-inflammatory cytokines and pro-oxidant profiles within the hypothalamic paraventricular nucleus of the spontaneously hypertensive rats (SHR). Basal values and variability of arterial pressure and heart rate and baroreflex sensitivity were measured in trained (T, low-intensity treadmill training) and sedentary (S) SHR at weeks 0, 1, 2, 4 and 8. Paraventricular nucleus was used to determine reactive oxygen species (dihydroethidium oxidation products, HPLC), NADPH oxidase subunits and pro-inflammatory cytokines expression (Real time PCR), p38 MAPK and ERK1/2 expression (Western blotting), NF-κB content (electrophoretic mobility shift assay) and cytokines immunofluorescence. SHR-S vs. WKY-S (Wistar Kyoto rats as time control) showed increased mean arterial pressure (172±3 mmHg), pressure variability and heart rate (358±7 b/min), decreased baroreflex sensitivity and heart rate variability, increased p47^phox^ and reactive oxygen species production, elevated NF-κB activity and increased TNF-α and IL-6 expression within the paraventricular nucleus of hypothalamus. Two weeks of training reversed all hypothalamic changes, reduced ERK1/2 phosphorylation and normalized baroreflex sensitivity (4.04±0.31 vs. 2.31±0.19 b/min/mmHg in SHR-S). These responses were followed by increased vagal component of heart rate variability (1.9-fold) and resting bradycardia (−13%) at the 4th week, and, by reduced vasomotor component of pressure variability (−28%) and decreased mean arterial pressure (−7%) only at the 8th week of training. Our findings indicate that independent of the high pressure levels in SHR, training promptly restores baroreflex function by disrupting the positive feedback between high oxidative stress and increased pro-inflammatory cytokines secretion within the hypothalamic paraventricular nucleus. These early adaptive responses precede the occurrence of training-induced resting bradycardia and blood pressure fall.

## Introduction

Baroreflex dysfunction, reflecting primarily an impairment in the vagal efferent component of the baroreceptor reflexes, is an important and common characteristic of arterial hypertension, closely related to sympathetic hyperactivity and activation of tissue and plasma renin-angiotensin systems (RAS), increased blood pressure variability, capillary rarefaction and end-organ damage, all of which lead to an increased mortality risk [1], [2]. Reduced baroreflex sensitivity (BrS) and heart rate (HR) variability are strong predictors of cardiac mortality and have been used as prognostic tools not only in hypertensive subjects, but also in post-myocardial infarction and renal failure patients [1], [3]–[5].

Several studies have shown that overactivity of tissue RAS increases angiotensin II availability, thereby activating nicotinamide adenine dinucleotide diphosphate (NADPH) oxidase and augmenting oxidative stress and the secretion of pro-inflammatory cytokines (PICs) [6]–[8]. Increased levels of PICs, such as tumor necrosis factor-alpha (TNF-α) and interleukin (IL)-6, were identified in the hypothalamic paraventricular nucleus (PVN) and other autonomic brain areas of hypertensive and heart failure rats [9]–[11]. Studies have also shown that PVN PICs increase sympathoexcitatory activity [9], [12] and that IL-6 microinjected into the nucleus tractus solitarii caused a pronounced reduction in BrS [13]. Central TNF-α inhibition reduced the expression of angiotensin type 1 receptor (AT_1_R), NADPH oxidase, TNF-α and IL-1β, increased the expression of neuronal and endothelial nitric oxide synthase, and reduced production of reactive oxygen species (ROS) in the PVN; central TNF-α inhibition simultaneously decreased plasma norepinephrine levels and reduced cardiac dysfunction in heart failure rats [14]. These findings suggest that PICs and oxidative stress modulate autonomic control.

Exercise training (T) is an adjuvant therapeutic tool in the management of hypertension. Experimental evidence has shown that T promotes several cardiovascular benefits [15]–[17] and improves both baroreflex function and autonomic control [18], [19]. T reduces RAS activity, oxidative stress and PICs secretion within autonomic brain areas of spontaneously hypertensive rats (SHR) [20], [21], which is the best experimental model of essential hypertension. T also downregulates AT_1_R and NADPH oxidase expression, decreases the pro-inflammatory profile in autonomic brain areas, increases baroreflex function, and decreases sympathetic activity in angiotensin II-infused rats and heart failure rabbits [22], [23]. In the SHR it was also shown that sinoaortic denervation blocked T-induced resting bradycardia, pressure fall and reduced sympathetic vasomotor variability [18] and markedly reduced T-induced plasticity in PVN preautonomic neurons [24]. Therefore, we hypothesized that training might improve BrS by altering pro-oxidant and pro-inflammatory profiles in the preautonomic PVN of SHR and that the improvement in BrS could contribute to blood pressure and HR reductions. We used SHR to investigate the effects of training on both hemodynamic parameters and oxidative/inflammatory status within the PVN, an important autonomic integrative area. Sedentary SHR and normotensive rats (WKY) were used as time controls. We have observed different temporal profiles for training-induced adjustments in ongoing experiments in our laboratory, so we also analyzed time-course changes during the study protocols.

## Methods

### Ethics Statement

All surgical procedures and experimental protocols were conducted in accordance with the National Institutes of Health Guide for the Care and Use of Laboratory Animals. The study was approved by the Institutional Animal Care and Use Committee of the Biomedical Sciences Institute of the University of Sao Paulo (CEUA no. 137).

### Animals and Training Protocols

Male 12-week old SHR and WKY were housed at a controlled room temperature (22°C) with a 12-hour dark-light cycle and free access to standard chow and water *ad libitum*. After an acclimatization period, SHR rats were preselected for their ability to walk/run on a treadmill (KT-300, Inbramed, Porto Alegre, Brazil; 5–10 sessions from 0.3 to 0.7 km/h, 0% grade, 5–10 min/day). Progressive maximal exercise tests on the treadmill (beginning at 0.3 km/h, with increments of 0.3 km/h every 3 min until exhaustion) [17], [18], [24] were used to allocate SHR with similar physical capacity to trained (T) and sedentary (S) protocols. Low-intensity T (50–60% of maximal exercise capacity, 0% grade, performed 5 days/week, 1 hour/day for 8 weeks) was similar to that used previously [17], [18], [24]. Maximal exercise tests were repeated at weeks 4 and 8 to adjust T intensity and compare the efficacy of T and S protocols, respectively. The rats allocated to the S protocol were handled every day and subjected once a week to a short period of mild exercise (5–10 min, 0.4–0.7 km/h) to keep S rats in conditions that approximated those experienced by the T group. Age-matched sedentary WKY rats were used as time controls.

### Hemodynamic Recordings and Baroreflex Testing

Experiments started with a group of 60 SHR and 20 WKY and were repeated to get the necessary *n* for functional and molecular studies. At established time points (weeks 0, 1, 2, 4 and 8 for the SHR-T and weeks 0 and 8 for SHR-S and WKY-S groups) some rats (∼10/sub-group) were anaesthetized (ketamine, 80 mg.kg^−1^ plus xylazine, 12 mg.kg^−1^, *ip*) for chronic implantation of catheters in the left femoral artery and vein [18], [24]. Cannulated rats were treated with antibiotic and analgesic and allowed to recover for 1 day. Arterial pressure (AP) and heart rate (HR) were continuously recorded in conscious freely moving rats on the following day, at least 24 hours after the last training session. Resting values of AP and HR were acquired on beat to beat basis for 30–40 min (computer, 2000 Hz sampling frequency, LabChart 2.0.1), as described previously [18], [24]. After baseline measurements, baroreflex function was determined by baroreceptors’ loading/unloading (phenylephrine and sodium nitroprusside *iv.*, 0.1–6.4 and 0.2–12.8 µg/kg, respectively, 100 µL bolus injection given in a random order); subsequent injections were not made until the recorded parameters had returned to pre-injection levels. Mean AP and HR values were measured before (control) and at the peak of each response. Baroreceptor reflex control of HR, determined for each rat, was estimated by the sigmoidal logistic equation fitted to data points, as described previously [25]. The equation linking HR responses to pressure changes was: HR = *P1*+*P2*/[1+e*^P3^* ^(BP–*P4*)^], where *P1* = lower HR plateau, *P2* = HR range, *P3* = the curvature coefficient and *P4* = BP_50_ (the value of blood pressure at half of the HR range). The average gain of baroreflex function (BrS) was calculated as BrS = –(*P2*×*P3*)/4.

### Power Spectral Analysis

Time series of resting systolic AP and pulse interval were used to calculate AP and HR variability, as well as their low (LF, 0.20–0.75 Hz) and high frequency components (HF, >0.75 Hz). The spontaneous baroreflex sensitivity (α index = LF of HR variability/HF of systolic AP) was also analyzed. These analyzes were performed by fast Fourier transformation, using the Matlab 6.0 software (Matworks, Natrick, MA USA) as described before [18].

### Tissue Sampling

At weeks 0, 1, 2, 4 and 8, after the functional measurements, rats were euthanized by an overdose of ketamine/xylazine anesthesia and subjected to transcardiac perfusion (∼5 min, Daigger pump, Vernon Hills, IL, USA) with PBS (0.01 M, pH 7.4, 20–30 ml/min) maintaining the perfusion pressure in a similar range as the recorded AP in the conscious animal. Rats were decapitated and the brain rapidly removed and transferred to a dry-ice box. Slices (800–1000 µm) were taken at the hypothalamic level and immediately frozen for bilateral punching of the PVN [24]. For immunofluorescence processing brains were fixed with 4% PFA infusion, post-fixed for 4 h at 4°C and cryoprotected (30% sucrose in 0.01 M PBS, 4°C) for 3–4 days [24]. Fresh samples and fixed brains were stored at −80°C until processing.

### Real Time RT-PCR

Total RNA was extracted using TRizol® reagent according to the manufacturer’s instructions (Invitrogen Life technologies, CA, USA) and measured by NanoDrop Spectrophotometer (Nano-Drop Techonologies, USA), with their integrity being checked by agarose gel electrophoresis. DNase (Invitrogen) was used to digest DNA and obtain pure RNA prior to RT-PCR reaction. RNA was primed with 0,5 µg/µl oligo DT to produce the first strand cDNA. Reverse transcription was made by M-MLV reverse transcriptase (Invitrogen) with the following incubation period: 5 minutes at 25°C, 60 minutes at 50°C, 15 minutes at 70°C and then hold on 4°C.

Standard curves for all analyzed genes were obtained with serial dilutions in order to determine the reactions’ efficiency. Inventoried Taqman gene assay (Applied Biosystem, Life Technologies CA, USA) was used for TNF-α (Rn99999017_m1), IL-6 (Rn00561420_m1), gp91^phox^ (Rn00576710_m1) and p47^phox^ (Rn00586945_m1) gene expression. Hypoxanthine-guanine phosphoribosil transferase (HPRT, Rn01527840_m1) was used as the reporter gene [24]. Real-time PCR amplification of targets mRNAs was performed using the ABI PRISM 7500 (Applied Biosystem, Life Technologies, CA, USA); each sample was analyzed in duplicate. The mRNA expression data were calculated by cycle threshold (Ct) values using the ΔΔCt method, and the results are expressed as fold increases, as described previously [24].

### Dihydroethidium (DHE) Oxidation

PVN punches were incubated with 0.5 ml of PBS/DTPA for 30 min. DHE oxidation was measured as previously described [26]. Briefly DHE was added to the buffer and the incubation carried out for 30 min at 37°C in a dark room. Samples were centrifuged and PBS/DTPA buffer was removed. Acetonitrile was added and the tissue sonicated in ice and centrifuged. The pellet was resuspended in lysis buffer, incubated in ice and centrifuged. Supernatants were dried under vacuum and pellets maintained at −20°C in the dark until analysis. Samples were resuspended in 80 µl of acetonitrile and injected into a HPLC system. Separation of DHE, 2-hydroxyethidium (EOH), and ethidium (E) was performed as described previously [26]. EOH/DHE and E/DHE ratios were obtained and normalized by protein concentration (measured by Bradfords method) in the pellet.

### Electrophoretic Mobility Shift Assay (EMSA)

PVN nuclear extracts were prepared as described previously [27]. Briefly, tissue was homogenized in a lysis buffer and incubated in ice for 15 min. NP-40 10% was added and samples were mixed and centrifuged (12,000 g for 30 s). Supernatants were kept for immunoblotting assay. The nuclei fraction was resuspended in the extraction buffer, incubated for 20 min on ice and centrifuged (12,000 g for 20 min). The supernatant was collected for measurement of protein concentration. EMSA for NF-κB was performed as described previously [28] with minor modifications. Double-stranded oligonucleotide containing the nuclear factor-kappaB (NF-κB) consensus sequence from Promega (5′-AGTTGAGGGGACTTTCCCAGGC-3′), was end-labeled using T4 polynucleotide kinase (Promega) in the presence of ^32^P dATP. Nuclear extracts (5 µg) were incubated with ^32^P-labeled NF-κB probe. The binding reaction was performed at room temperature for 30 min in the reaction buffer containing 50 mM Tris-HCl pH 7.5, 250 mM NaCl, 5 mM MgCl2, 2.5 mM EDTA, 20% glycerol, 0.25 µg/µL of poly (dI-dC) and 2.5 mM dithiothreitol. DNA protein complexes were separated by electrophoresis through a 6% acrylamide:bis-acrylamide (37.5∶1) gel in TBE (45 mM Tris, 45 mM Boric Acid, 0.5 mM EDTA) for 2 h at 150 V. Gels were vacuum dried for 1 h at 80°C and exposed to X-ray film at −80°C.

### Western Blotting Analysis

Immunoblottings were performed as described previously [27]. Briefly, the proteins were size-separated in 10% SDS-PAGE and blotted into a PVDF membrane (Millipore). Primary antibodies used were phospho-ERK1/2 (1∶1000, Cell Signaling Technology), total-ERK1/2 (1∶2000, Santa Cruz Biotechnology), phospho-p38 (1∶1000, Cell Signaling Technology), total-p38 (1∶2000, Santa Cruz Biotechnology) and β-actin (1∶2000, Sigma). HRP-conjugated secondary antibody (1∶2000, Sigma) was applied and blots developed using an ECL kit (Millipore). Data analyses were performed using Image J software (NIH, USA).

### Immunofluorescence

Sequential hypothalamic coronal sections (30 µm, −1.80 to −2.12 caudal to the Bregma, 3 rats/subgroup) were cut with a cryostat (Leica CM 1850; Nussloch, Germany) and collected in tissue culture wells with 0.1 M PB. Free-floating sections were pretreated with 1% H_2_O_2_ for 30 min, washed with 0.1 M PB for 30 min and incubated with 2% normal donkey serum for 30 min. For immunofluorescence reactions, the sections were incubated overnight with primary antibody (polyclonal goat anti-TNF-α, 1∶40 dilution; R&D Systems, USA), followed by a 2-hours incubation with secondary antibody (donkey anti-goat labeled with Alexa 594; Jackson ImmunoResearch Laboratories, West Grove, PA, USA). Four to six slices were placed in each slide and mounted with a coverslip and Vectashield. Negative controls omitted the 1^ary^ or the 2^ary^ antibody. The sections were examined to localize PVN (Leica DMLB, Wetzlar, Germany). Imaging analysis was performed with Image ProPlus software (Media Cybernetics, Silver Spring, MD).

### Statistical Analysis

The results are expressed as mean ± SEM. The comparison between groups (WKY-S, SHR-S and SHR-T) at different time-points (weeks 0 and 8) was made by two-way ANOVA. The effect of training in the SHR-T group during the 8-week protocol was analyzed by the factorial one-way ANOVA (Statistica 7.0, Vince Stat Software). Fisher’s was used as the *post hoc* test. Differences were considered significant at *P*<0.05.

## Results

### Effects of Training on Treadmill Performance

At the beginning of experiments SHR exhibited a better treadmill performance, as measured by the duration of maximal exercise tests (798±100 s *vs*. 350±137 s in the WKY). SHR submitted to low intensity T exhibited a significant increase in the aerobic capacity at week 4 (1107±157 s), with a further increment at week 8 (1352±154 s). In contrast, SHR-S group showed a significant decrease on treadmill performance during the experimental period (479±159**s at the 8^th^ week). Therefore at the end of the experimental period SHR-T exhibited a large increment on the aerobic capacity (2.8-fold increase over SHR-S values). The WKY-S group did not show any change in the duration of maximal exercise tests during the 8 weeks-protocol.

### Sequential Effects of Training on AP, HR and BrS

Table 1 shows time-course changes of resting mean AP and HR during the T protocol. SHR had well-established hypertension at baseline, demonstrated by higher MAP and HR levels than age-matched WKY. The SHR-T group showed a significant decrease in resting HR at week 4 (−5%), with a further decrease at week 8 (−13%). These changes were accompanied by a significant decrease in MAP only at week 8 (−9%). At the end of protocols SHR-T group maintained an elevated AP, but basal HR values were similar to the WKY group (Table 1).

**Table 1:** Basal values of arterial pressure and heart rate with respective low (LF) and high frequency (HF) components in trained (T) and sedentary (S) SHR and sedentary WKY groups at the different time-points.

	Arterial Pressure			Heart Rate or Pulse Interval		
	Basal MAP (mmHg)	LF of SAP (mmHg^2^)	HF of SAP (mmHg^2^)	Basal HR (b/min)	LF (ms^2^)	HF (ms^2^)
**SHR groups**						
**S_0_ = T_0_** (n = 11)	172±3*	3.30±0.19*	1.61±0.17*	358±7*	1.12±0.13	3.47±0.30*
**T_1_** (n = 12)	168±3	2.85±0.24	1.59±0.24	348±7	0.73±0.08	3.66±0.35
**T_2_** (n = 13)	168±3	2.80±0.22	1.60±0.13	340±8	0.88±0.12	4.51±0.55
**T_4_** (n = 11)	174±2	2.49±0.24‡	1.75±0.14	339±5‡	1.23±0.15	4.92±0.33‡
**T_8_** (n = 13)	156±5*† ‡	2.37±0.23*† ‡	2.29±0.42*† ‡	313±7† ‡	1.31±0.23	6.66±1.57† ‡
**S_8_** (n = 12)	173±6*	4.35±0.44*‡	1.70±0.12	361±13*	1.14±0.22	4.02±0.33*
**WKY groups**						
**S_0_** (n = 11)	118±2	1.13±0.11	1.04±0.08	307±6	1.39±0.33	4.80±0.37
**S_8_** (n = 12)	117±2	1.13±0.09	1.45±0.12	321±9	1.09±0.14	5.61±0.51

Values are means ± SEM; *n* is the number of rats in each subgroup. MAP, mean arterial pressure; SAP, systolic arterial pressure; HR, heart rate. Significances (*P<0.05*) are: *vs time-paired WKY,

† *vs* respective sedentary control,

‡ *vs* week zero (T_0_ or S_0_).

**Figure 1:**
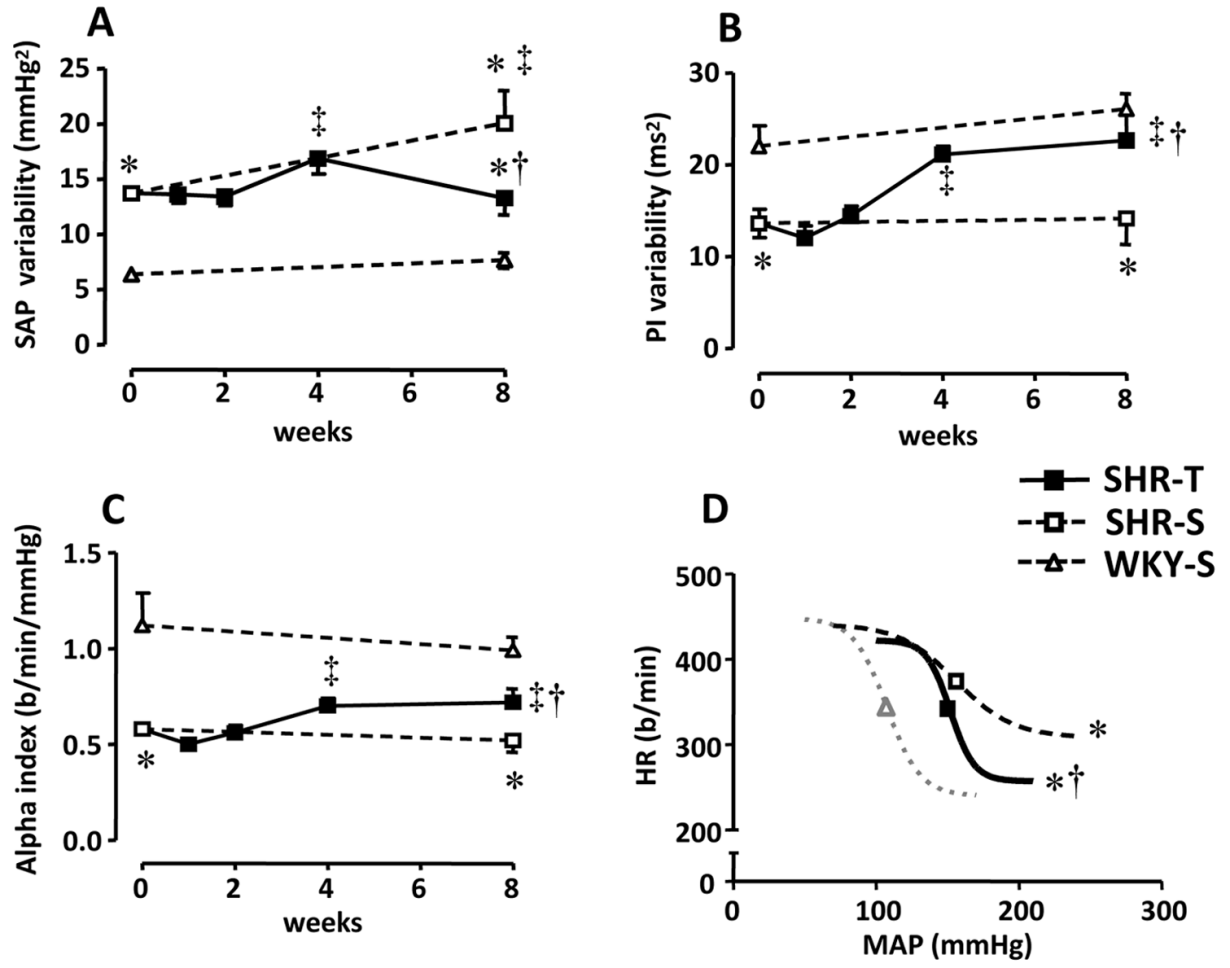
Sequential changes of systolic arterial pressure variability (SAP, panel A), pulse interval variability (PI; panel B), spontaneous baroreflex sensitivity (indicated by the alpha index; panel C) induced by training (T) or sedentary (S) protocol in hypertensive (SHR) and normotensive (WKY) rats. Panel D shows sigmoidal logistic function curves of baroreceptor reflex control of HR in WKY-S, SHR-S and SHR-T at the end of the protocols. *n* = 11–13 rats/subgroup. Significances (*P<0.05*) are: * *vs* time-paired WKY, † *vs* respective sedentary control, and ‡ *vs* week zero (T_0_ or S_0_).

SAP variability was higher and PI variability was lower in SHR-S_0_ compared to WKY-S_0_ (Figures 1A and 1B). In the SHR-S group, SAP variability increased (+46% from S_0_ to S_8_, p<0.05< Figure 1A), while PI variability was unchanged during the 8-week protocol (Figure 1B). The SHR-T group exhibited a significant increase in PI variability (from 13.6±1.5 ms^2^ at T_0_ to 21.1±0.9 ms^2^ at T_4_). This increase was maintained through week 8 and accompanied by a significant high frequency (HF) component increase at week 4 (Table 1). Training also eliminated the increased SAP variability observed in SHR-S_8_ (Figure 1A), which was accompanied by low frequency (LF) reduction and HF increase (−28% and +42% respectively, for SHR-T_8_ compared to SHR-S_8,_ Table 1). Spontaneous BrS, estimated by the α-index of LF, was improved by training (0.71±0.04 b/min/mmHg at T_4_–T_8_ *vs.* 0.58±0.03 b/min/mmHg at T_0_; Figure 1C). However, training normalized the stimulated BrS at weeks 1 to 2 (Table 2), as indicated by the increased HR range (there was a significant downward displacement of the lower plateau) and the steeper inclination of the curve in the middle range (see parameters of logistic function curve in Table 2). These changes were maintained through week 8. At the end of protocols, the baroreflex function curve of the SHR-T group was similar to that exhibited by the WKY group, but still displayed higher levels of blood pressure (Figure 1D).

**Table 2:** Parameters of logistic function curve of stimulated baroreflex control of heart rate (HR) in trained (T) and sedentary (S) SHR and sedentary WKY groups at the different time-points.

	Baroreflex Control of Heart Rate			
	Lower plateau (b/min)	HR range (B/min)	BP50 (mmHg)	Gain (b/min/mmHg)
**SHR groups**				
**S_0_ = T_0_** (n = 11)	311±5*	122±12*	159±13	2.31±0.19*
**T_1_** (n = 12)	277±14‡	179±13‡	157±4	2.61±0.12
**T_2_** (n = 12)	305±9	159±9	152±4	4.04±0.31‡
**T_4_** (n = 11)	288±9‡	168±10‡	167±3	4.00±0.55‡
**T_8_** (n = 12)	259±12† ‡	169±9† ‡	152±2	4.04±0.31† ‡
**S_8_** (n = 11)	288±13*	145±11*	159±5	2.00±0.21*
**WKY groups**				
**S_0_** (n = 11)	241±13	200±18	110±3	3.58±0.32
**S_8_** (n = 11)	240±15	199±18	107±5	3.89±0.51

Values are means ± SEM; *n* is the number of rats in each subgroup. BP50, blood pressure at the mid-range. Significances (*P<0.05*) are: * *vs* time-paired WKY,

† *vs* respective sedentary control,

‡ *vs* week zero (T_0_ or S_0_).

### Sequential Effects of Training on PVN NADPH Oxidase Expression and ROS Generation

**Figure 2:**
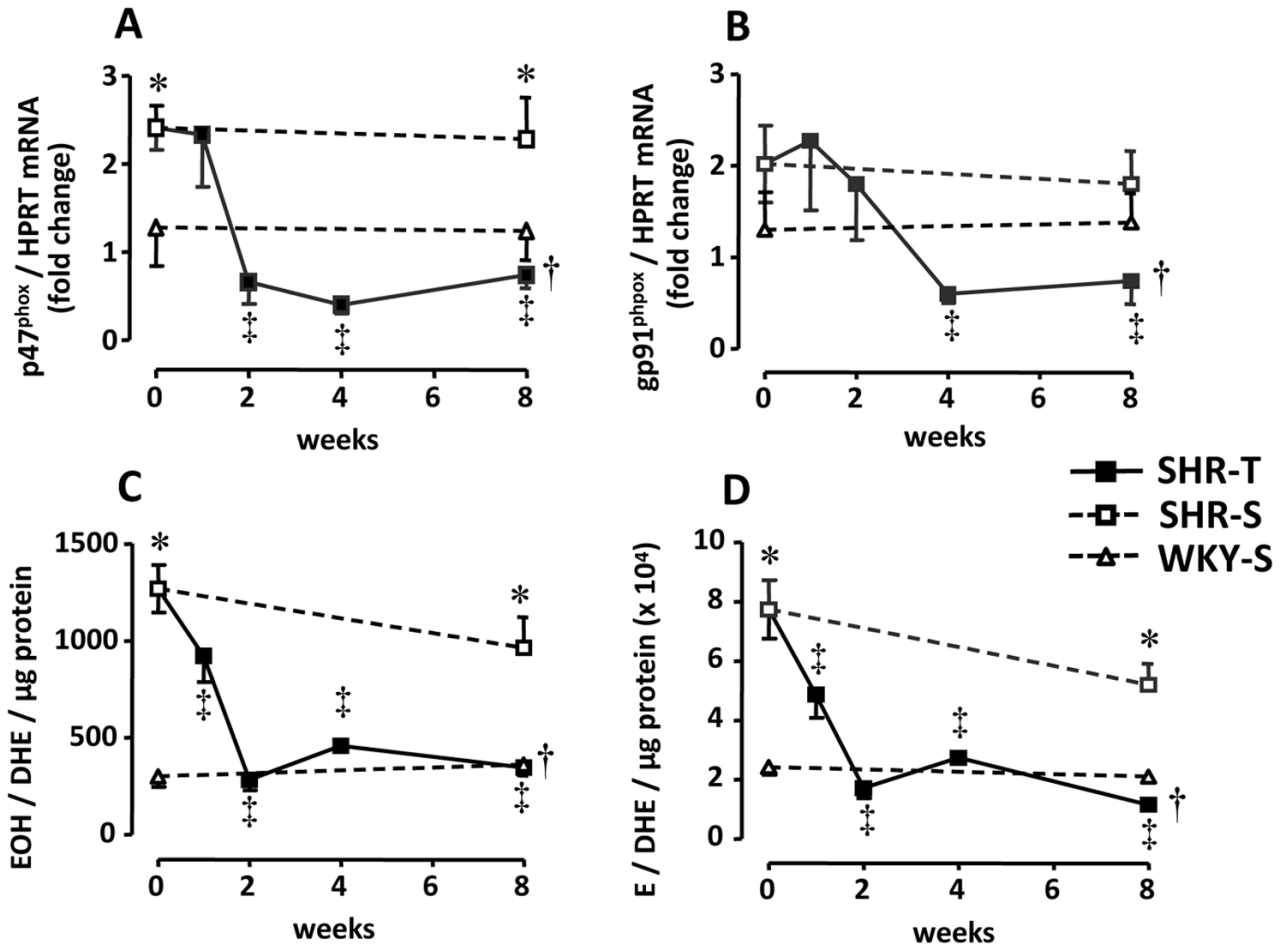
Sequential changes of p47^phox^ and gp91^phox^ subunits of the NADPH oxidase (panels A and B, respectively, *n* = 6–8 rats/subgroup) and of EOH/DHE and E/DHE ratios (panels C and D, respectively, *n* = 5 rats/subgroup) within the PVN induced by training (T) or sedentary (S) protocol in hypertensive (SHR) and normotensive (WKY) rats. Significances (*P<0.05*) are: * *vs* time-paired WKY, † *vs* respective sedentary control, and ‡ *vs* week zero (T_0_ or S_0_).

Compared to WKY-S_0_, SHR-S_0_ displayed increased mRNA expression of NADPH oxidase subunits (+83% for p47^phox^, p<0.05, and +67% for gp91^phox^, p>0.05; Figures 2A and 2B, respectively). Training was accompanied by large decreases in the expression of both subunits: p47^phox^ expression was markedly reduced at week 2 (from 2.34±0.17 AU at T_0_ to 0.66±0.25 AU at T_2_), while gp91^phox^ was significantly decreased at week 4 (from 2.17±0.22 AU at T_0_ to 0.60±0.10 AU at T_4_). These reductions were maintained through week 8. Accordingly, superoxide anion generation (Figure 2C) and the production of other reactive species in the PVN (Figure 2D), which were significantly increased in SHR-S_0_ compared to WKY-S_0_, showed a marked reduction at week 2 (−78% for both EOH/DHE and E/DHE ratios, expressed per µg of protein). ROS levels remained similar to those presented by normotensive rats from weeks 2 through 8. Neither sedentary control group showed significant changes in p47^phox^ or gp91^phox^ mRNA expression or in EOH/DHE and E/DHE ratios during the 8-week protocol.

### Sequential Effects of Training on PVN MAPK Activity and NF-κB Expression

**Figure 3:**
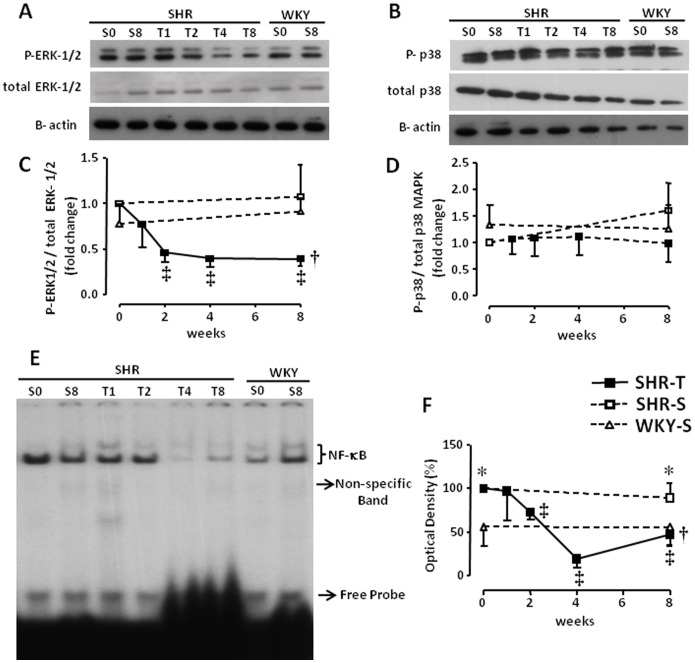
Sequential changes of phospho-ERK-1/2/total ERK-1/2 (panels A and C) and phospho-p28/total p28 (panels B and D) protein expression within the PVN induced by training (T) or sedentary (S) protocol in hypertensive (SHR) and normotensive (WKY) rats. Changes in NF-κB during S and T protocols are presented on panels E (gel shift) and F (measurement of optical density). *n* = 4–5 rats/subgroup. Significances (*P<0.05*) are: * *vs* time-paired WKY, † *vs* respective sedentary control, and ‡ *vs* week zero (T_0_ or S_0_).

The effects of aerobic training on the expression of MAPK subfamilies are depicted in Figure 3. SHR-S_0_ and WKY-S_0_ exhibited similar PVN expression of phospho/total ERK1/2 and p38 MAPK (Figures 3A and 3C, and 3B and 3D, respectively). Training, however, markedly decreased the phosphorylation of ERK1/2 from week 2 to week 8 (0.42±0.09 AU; corresponding to a reduction of 58%), attaining values lower than those presented by the WKY group (0.91±0.15 AU; Figure 3C). In contrast, training did not change the phosphorylation of p38 MAPK (Figures 3B and 3D). NF-κB binding to DNA was increased in SHR-S_0_ compared to WKY-S_0_, but it was significantly reduced by training from week 2 to week 8 (Figures 3E and 3F). NF-κB expression in SHR-T_8_ was similar to that of WKY. Again, no changes in the ratio of phosphor-ERK1/2/total protein or NF-κB binding to DNA were observed in SHR-S or WKY-S groups during the 8-week protocol.

### Sequential Effects of Training on PVN PICs Expression

**Figure 4:**
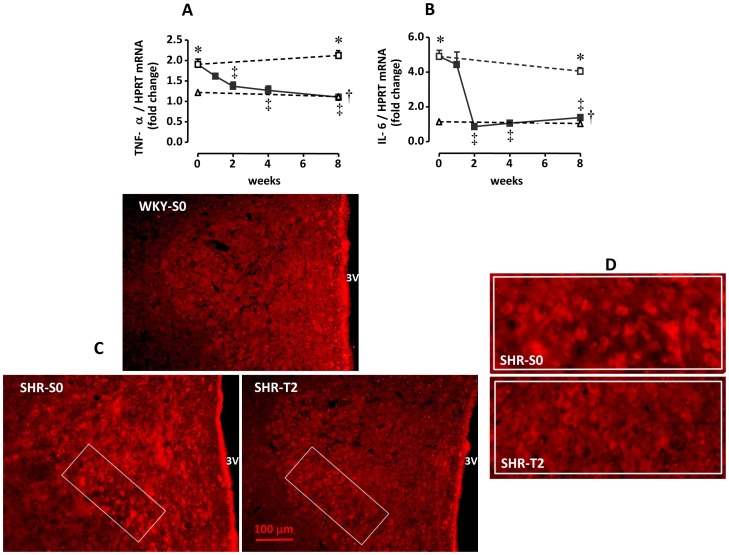
Sequential changes of TNF-α (panel A) and IL-6 (panel B) mRNA expression within the PVN induced by training (T) or sedentary (S) protocol in hypertensive (SHR) and normotensive (WKY) rats. *n* = 6–8 rats/subgroup Significances (*P<0.05*) are: * *vs* time-paired WKY, † *vs* respective sedentary control, and ‡ *vs* week zero (T_0_ or S_0_). The bottom panels on C and D (magnified images of the ventromedial subnucleus of medial PVN, a preautonomic area) depict the marked reduction in TNF-α immunoreactivity after 2 weeks of training in the PVN of a hypertensive rat (SHR-T_2_) compared with a sedentary control (SHR-S_0_), which shows an intense fluorescent signal. The fluorescent signal in a normotensive control is also shown (WKY-S_0_, panel C) for comparison. 3V, 3^rd^ ventricle.

TNF-α and IL-6 mRNA expression were higher in SHR-S_0_ compared to WKY-S_0_ (Figures 4A and 4B). Training significantly decreased PICs expression in the PVN at week 2 (TNF-α: from 1.91±0.34 AU at T_0_ to 1.27±0.28 AU at T_2_; IL-6: from 4.91±0.85 AU at T_0_ to 1.07±0.19 AU at T_2_, corresponding to 34% and 78% reductions, respectively; p<0.05). No further changes were observed through week 8. PICs expression was not changed in SHR or WKY sedentary controls (Figures 4A and 4B). The effect of training on PVN TNF-α was confirmed by immunofluorescence assay; the densest immunoreactivity exhibited by SHR-S_0_ was largely decreased after 2 weeks of T to a value similar to that of WKY-S_0_ (Figures 4C and 4D). The training-induced decrease in both TNF-α and IL-6 expression in the PVN correlated with training-induced increases in BrS (Y_TNF-α_ = −0.56x+4, r = −0.361; p = 0.017; and Y_IL-6_ = −0.39x+4, r = −0.511; p = 0.001, respectively).

## Discussion

**Figure 5:**
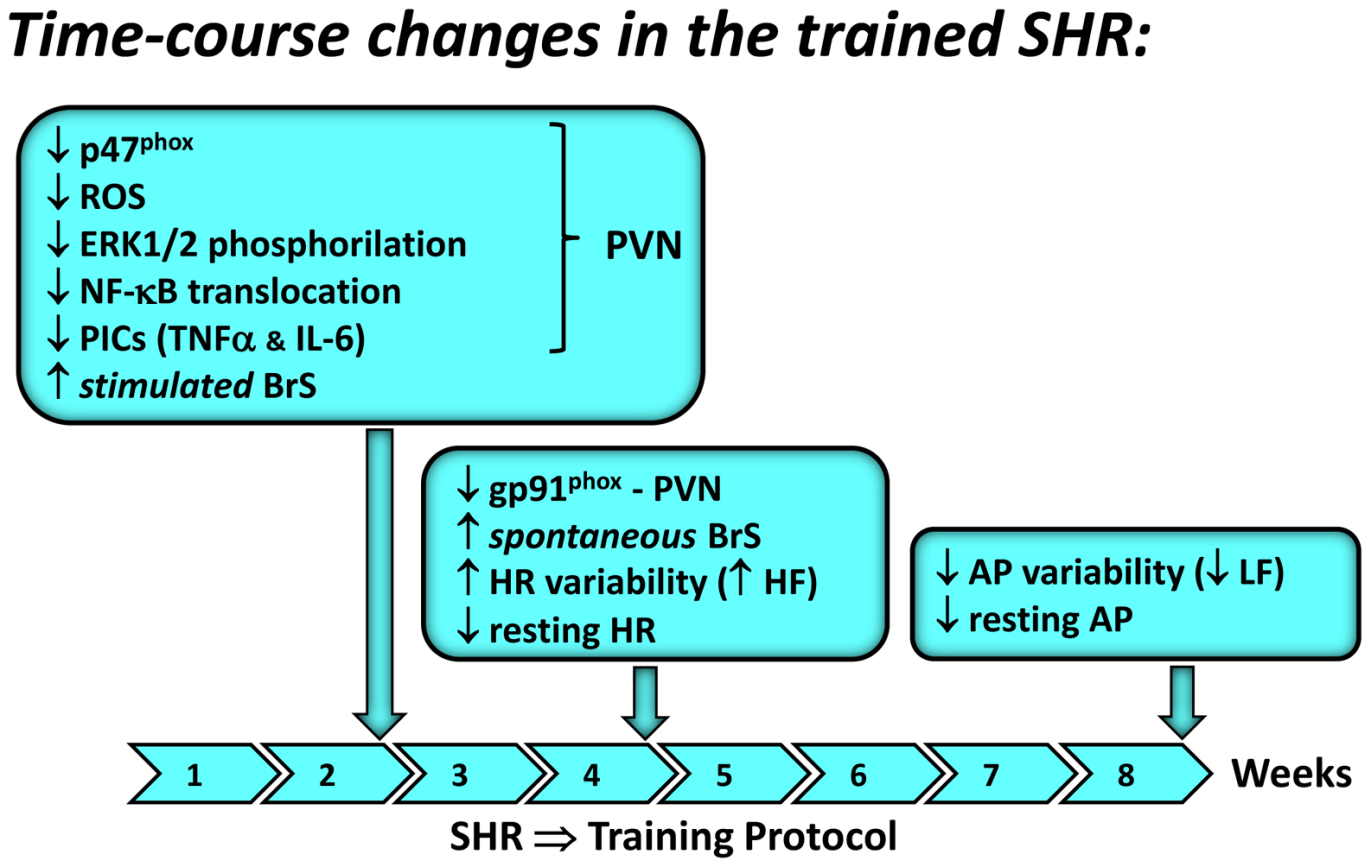
Scheme depicting time-course changes in PVN signaling and functional responses in the trained SHR during the 8-week protocol.

This study confirms the beneficial effects of low-intensity exercise training to improve cardiovascular control in SHR. Our data also reveal three original observations (Figure 5). First, training-induced benefits appear sequentially. They are preceded by an early improvement in baroreceptor reflex control of HR, which occurs immediately before or simultaneously with the augmentation of vagal control of the heart and the reduction of resting HR; this is followed by a late decrease in pressure, which is associated with a reduced LF component of pressure variability. Second, these responses are preceded by prompt and sustained normalization of NADPH oxidase and ROS content, suppression of ERK1/2 phosphorylation, marked reduction of NF-κB translocation to the nucleus, and normalization of PICs within the PVN, an important autonomic integrative brain area. Third, observed time-course changes suggest that training-induced reduction of pro-oxidative and pro-inflammatory profiles in the PVN drive baroreflex improvement, which controls the appearance of resting bradycardia and contributes to the late decrease in blood pressure.

Since the 1960s, baroreflex dysfunction has been considered a hallmark for hypertension [29]. More recently, reduced BrS has been considered an independent risk factor for mortality and major adverse cardiovascular events in hypertensive subjects [4], post-myocardial infarction patients [1] and patients with renal failure [3]. SHR also exhibits impaired baroreceptor reflex function [18], [30]. Accordingly, data from the present study confirmed decreased baroreflex function in sedentary SHR (*vs.* sedentary WKY), characterized by upward displacement of the lower HR plateau, a smaller HR range, reduced sensitivity and rightward displacement of the baroreflex curve toward increased pressure levels. Although the ability of exercise training to improve baroreflex function in hypertensive and myocardial infarction patients is already known [1], [18], [30] the data presented here show for the first time that normalization of baroreceptor reflex control of HR in hypertensive individuals is a prompt and sustained response, beginning in weeks 1–2 and continuing through week 8. This normalization might contribute to the other cardiovascular training-induced adjustments, particularly the improvement of vagal control of the heart (T_4_) and the reduction of vasomotor variability (T_8_). Indeed, previous studies proved that sinoaortic denervation blocked the positive correlation between LF reduction and training-induced decreases in pressure observed in SHRs [18]. It is important to note that, after sinoaortic denervation, there was no resting bradycardia and no blood pressure reduction in the trained SHR [18], [24], [31], [32] confirming the decisive role of baroreflex to drive training-induced cardiovascular adjustments.

Experimental evidence has also shown altered PVN function in hypertension. High pressure levels in SHR, renal and angiotensin II-induced hypertension are mediated by AT_1_R [11], [21], [22], [33], which activates NADPH oxidase and increases oxidative stress [7], [10], [21], [22], [33]. In the presence of increased oxidative stress, ERK1/2 (but not p38 MAP kinase) is activated [11] to increase NF-κB translocation to the nucleus [10], which upregulates PVN expression of TNF-α and other PICs [10], [21]. Within the PVN, TNF-α and IL-1β increased renal sympathetic nerve activity and blood pressure [12] while chronic blockade of TNF-α [34] and NF-κB [9], [10] inhibited hypertension-induced cardiovascular effects. It should be noted that NF-κB blockade given intraperitoneally was not effective [9]. These observations reinforce the link between PVN oxidative and inflammatory profile and autonomic dysfunction in hypertension. The present study confirmed these observations in sedentary SHR. In the brain, ERK1/2 also activates CREB transcription factor, which upregulates AT_1_R gene expression [35]. In endothelial cells, TNF-α phosphorylates the p47^phox^ NADPH subunit to increase ROS production [36]. These effects might give rise to positive feedback loops that perpetuate TNF-α and IL-6 generation in the PVN and exacerbate autonomic dysfunction in SHR. In fact, IL-6 reduced BrS when microinjected into the nucleus tractus solitarii [13]. Interestingly, a short period of exercise training is sufficient to promptly reduce and normalize oxidative stress and PICs expression in the PVN. Chronic exercise attenuates the RAS vasoconstrictor axis, the pro-inflammatory profile and ROS content in the PVN in hypertensive [21], [22] and heart failure rats [9] and rabbits [23], but our data is the first to examine time-course changes that reveal normalized expression of NADPH oxidase subunits, restored ROS production, decreased ERK1/2 phosphorylation, reduced NF-κB activation and a normalized pro-inflammatory profile in the PVN within 2 weeks of training and maintenance of these values through the end of the protocol. Notice that these training-induced responses occurred in the absence of pressure fall. Together these effects suggest a prompt exercise-induce reduction in the activity of both RAS and downstream pathways within the PVN of hypertensive rats submitted to a low-intensity training protocol.

Two other important findings in this study are: 1) training-induced normalization of baroreceptor reflex control of HR and training-induced improvement of intracellular signaling within PVN neurons occurred simultaneously; 2) BrS was negatively correlated with both TNF-α and IL-6 content in the PVN. Therefore, reductions of pro-inflammatory and pro-oxidant profiles in the trained SHR are crucial mechanisms driving, by the second week of training, the normalization of baroreflex control even in persistent hypertension. Previously, we showed an increased expression of oxytocinergic preautonomic PVN neurons with a denser projection to the solitary-vagal complex in trained SHR, and these plastic changes were accompanied by improved vagal outflow, increased BrS and reduced resting HR [24], [25], [32], [37], [38]. Also, oxytocin attenuated NADPH-dependent activity and IL-6 secretion in macrophages and vascular cells [39]. Taken together, these findings suggest that training-induced improvement of cardiovascular control by oxytocinergic preautonomic neurons could be mediated through the normalization of oxidative and inflammatory profiles within the PVN. The results of the present study support this theory. Importantly, cardiovascular training effects were completely blocked by sinoaortic denervation [18], [24], [32] reinforcing the relationship between PVN adaptive changes, improved baroreflex function, augmented vagal outflow to the heart and improved cardiovascular control in the trained SHR here observed. Training-induced improvement in reflex bradycardia (downward displacement of lower HR plateau, which is a vagal effect) is paramount, since the ATRAMI and EVABAR trials showed that reduced vagal reflexes during pressure increases was the major cause of adverse cardiovascular events in myocardial infarction and hypertensive patients [1], [4].

It should be noted that the present study did not include a trained WKY group since pilot experiments did not show important effects of training on PVN oxidative and inflammatory profile in the normotensive strain. Another limitation of this study is that molecular analysis of oxidative and inflammatory profile was restricted to the PVN and did not include other brain regions implicated in the autonomic control and in the pathogenesis of hypertension. Also a caveat to be taken into consideration is that tissue punches used for several analyses included different cell types with different projections and different functions.

In conclusion, data of the present study associating hemodynamic recordings, baroreflex testing and molecular biology analyses in the same animals indicate that exercise training disrupts the positive feedback between high oxidative stress and increased pro-inflammatory profile in the PVN, baroreflex dysfunction and high pressure levels exhibited by sedentary SHR. Importantly, exercise restores BrS within the first 2 weeks of the training protocol, independent of baseline blood pressure levels. Data also indicate that normalization of baroreflex control is the first adaptive response of the cardiovascular system to exercise training and precedes the occurrence of resting bradycardia and reduced blood pressure.
